# Smoking may compromise physical function long before it kills you

**DOI:** 10.3389/fpubh.2023.1261102

**Published:** 2023-11-08

**Authors:** Dana A. Glei, Maxine Weinstein

**Affiliations:** Center for Population and Health, Georgetown University, Washington, DC, United States

**Keywords:** smoking, physical function, physical limitations, midlife, age variation, United States

## Abstract

**Introduction:**

Although prior research has demonstrated an association between smoking and worse physical function, most of those studies are based on older people and do not evaluate whether the age-related increase in physical limitations differs by smoking history. We quantify how the magnitude of the smoking differential varies across age.

**Methods:**

This cohort study comprised a national sample of Americans aged 20–75 in 1995–1996, who were re-interviewed in 2004–2005 and 2013–2014. Our analysis was restricted to respondents who completed the self-administered questionnaires at Wave 1 (*N* = 6,325). Follow-up observations for those respondents were included if they completed the self-administered questionnaires at Wave 2 (*N* = 3,929) and/or Wave 3 (*N* = 2,849). The final analysis sample comprised 13,103 observations over a follow-up period of up to 19 years (1995–2014). We used a linear mixed model to regress physical limitations on smoking status at baseline adjusted for sex, age, race, socioeconomic status, alcohol abuse, drug abuse, and obesity with an interaction between age and smoking to test whether the age pattern of physical limitations differed by smoking history. Additional models incorporated measures of smoking duration and intensity.

**Results:**

In the fully-adjusted model, smokers exhibited a steeper age-related increase in physical limitations than never smokers. Thus, the disparities in physical limitations by smoking status widened with age but were evident even at young ages. The estimated differential between heavy smokers and never smokers rose from 0.24 SD at age 30 to 0.49 SD at age 80. At younger ages, heavy smokers who quit recently fared worse than current light smokers and not much better than current heavy smokers.

**Discussion:**

We know smoking is bad for our health, but these results reveal that differences in physical limitations by smoking history are evident even at ages as young as 30. Physical limitations that emerge early in life are likely to have an especially large impact because they can jeopardize health for decades of remaining life. Smoking probably will not kill you at young age, but it may compromise your physical function long before it kills you. Just do not do it.

## 1. Introduction

We all know that smoking is bad for our health. What most of us do not appreciate is just how soon the adverse consequences emerge. Many smokers start early in life; As we show here, it turns out that it’s a short trip to physical limitations. Many prior studies have demonstrated that smoking is associated with worse physical function (1–5). However, most prior studies of the association between smoking and physical function are based on older people.

A few studies included individuals younger than 40 (6–8), but they did not evaluate whether the age-related increase in physical limitations differed by smoking status. One study included women as young as 18, with models fit separately for younger, middle-aged, and older cohorts (9). Their results suggested that the effect of smoking may be larger in midlife than at younger ages, but they did not formally evaluate whether the association between smoking and physical function differed across the full age range.

Although people generally adopt smoking early in life, many of the health consequences do not emerge until later in life. The question arises: how early in life does a smoking differential in physical function become evident? Here, we evaluate differences in physical limitations by smoking history among a national sample of Americans aged 20–75 in 1995–1996, who were re-interviewed in 2004–2005 and 2013–2014. We investigate whether the age-related increase in physical limitations differs by smoking history and quantify how the magnitude of the smoking differential varies across age.

## 2. Materials and methods

### 2.1. Data

This cohort study comprised data from Waves 1, 2, and 3 of the Midlife in the United States (MIDUS) study. At Wave 1 (fielded January 1995–September 1996), MIDUS targeted non-institutionalized, English-speaking adults aged 25–74^1^ in the contiguous United States (10). See section 1.1 of Supplementary material for details regarding sampling. At Wave 2 (fielded January 2004–August 2005), a follow-up telephone interview was conducted with 4,963 of the original MIDUS cohort (75% of survivors), 4,041 (81%) of whom also completed the mail-in self-administered questionnaires. At Wave 3 (fielded May 2013–June 2014), the MIDUS cohort was re-contacted for a second follow-up telephone interview (which was completed by *N* = 3,294, 56% of survivors) and self-administered questionnaires (*N* = 2,924, 89% of those who completed the phone interview). The datasets analyzed for this study can be found in the Inter-university Consortium for Political and Social Research (https://www.icpsr.umich.edu/icpsrweb/ICPSR/studies/2760; https://www.icpsr.umich.edu/web/NACDA/studies/4652; https://www.icpsr.umich.edu/web/NACDA/studies/36346).

Our analysis was restricted to respondents who completed the self-administered questionnaires at Wave 1 (*N* = 6,325). We included follow-up observations for those respondents if they completed the self-administered questionnaires at Wave 2 (*N* = 3,929) and/or Wave 3 (*N* = 2,849). Thus, the final analysis sample comprised 13,103 observations (mean follow-up 9.9 years, maximum 19.3 years). Respondents who died before Wave 2 or were lost-to-follow-up at Waves 2 and 3 were observed only at baseline. Those who died between Waves 2 and 3 or were lost-to-follow-up at Wave 3 were observed only at Waves 1 and 2. Among those who survived to June 2014 (when Wave 3 fieldwork ended), the mean follow-up was 11.2 years.

MIDUS conformed to the principles embodied in the Declaration of Helsinki and was approved by the Educational and Social/Behavioral Science Institutional Review Board at University of Wisconsin, Madison (#SE-2011-035).

### 2.2. Measures

#### 2.2.1. Physical limitations

Self-reported physical limitations were measured at all three waves. Respondents were asked, “How much does your health limit you in doing each of the following? Lifting or carrying groceries; climbing several flights of stairs; bending, kneeling, or stooping; walking more than a mile; walking several blocks; walking one block; vigorous activity (e.g., running, lifting heavy objects); moderate activity (e.g., bowling, vacuuming).” The response categories for each of the eight physical tasks were coded on a four-point scale (0 = not at all, 1 = a little, 2 = some, 3 = a lot). Based on Long and Pavalko (11), we constructed an index by summing the eight items (potential range 0–24), adding a constant (0.5), and taking the logarithm of the result, which allows for relative rather than absolute effects. Then, we standardized the resulting scores based on the pooled distribution across all three waves.

#### 2.2.2. Smoking

Smoking history (never smoker, former smoker, current smoker) and other smoking-related measures were measured only at baseline to avoid potential endogeneity (i.e., some respondents may have quit smoking because of serious health problems). We measured smoking duration by subtracting the age when the respondent started “smoking regularly (i.e., at least a few cigarettes every day)” from the age at which s/he quit smoking (for former smokers) or their current age (for those still smoking). For former smokers, we categorized the number of years since they quit smoking into three groups (<5 years prior to baseline, 5–9 years, and 10 or more years). Smoking intensity was represented by the average number of cigarettes smoked per day “during the 1 year in your life when you smoked most heavily.” Pack-years was computed as duration multiplied by intensity.

#### 2.2.3. Control variables

We controlled for sex, age, race, socioeconomic status, alcohol abuse, drug abuse, and obesity. Age was a time-varying covariate updated at Waves 2 and 3, while the other covariates were measured at baseline.

A composite measure of relative socioeconomic status was based on six variables: educational attainment and occupational socioeconomic index of the respondent (and spouse/partner, if applicable), annual household income, and current net assets of the respondent and spouse. We standardized all six items and calculated the mean across relevant items (e.g., six items if married/partnered and both respondent and spouse/partner were ever been employed; three items if not married/partnered and respondent was never employed; Cronbach’s *α* = 0.75). Then we converted the composite score to a percentile rank representing the individual’s position within the baseline distribution.

Alcohol abuse was a dichotomous measure based on four items from the Michigan Alcohol Screening Test (12). The respondent was coded as exhibiting alcohol abuse if s/he reported any of four alcohol-related problems in the past 12 months: alcohol use in hazardous situations; emotional or psychological problems as a result of alcohol use; strong desire or urge to use alcohol; a great deal of time using/recovering; using more to get the same effect.

Drug abuse was a binary measure based on the World Health Organization Composite International Diagnostic Interview short-form drug dependence scale (13). The respondent was coded as exhibiting drug abuse if s/he reported any of seven drug-related problems in the past 12 months: role interference as a result of use; use in hazardous situations; emotional or psychological problems as a result of use; strong desire or urge to use drugs; a great deal of time using/recovering; using more or for longer than intended; using more to get the same effect.

We created an index of obesity based on five self-reported items: current body mass index (BMI), BMI 1 year prior to baseline, waist circumference, hip circumference, and a subjective question asking the respondent whether s/he considers him/herself overweight. The two BMI measures were standardized based on the distribution of the full sample at baseline. Because of sex-differences in waist circumference, hip circumference, and the subjective evaluation of being overweight, we standardized those three variables based on the sex-specific distribution. All five items loaded well (≥0.68) on one factor (eigenvalue = 3.7). We computed the obesity index as the average across the five standardized items (Cronbach’s *α* = 0.93).

See section 1.2 of Supplementary material for more details regarding construction of the measures. Table 1 shows descriptive statistics for the analysis variables.

**Table 1:** Descriptive statistics for analysis variables by survey wave.

Variable	Wave 1 (1995–96)	Wave 2 (2004–05)	Wave 3 (2013–14)
Female, N (%)^a^	3,321 (52.5)	2,180 (55.5)	1,575 (55.3)
Age (20–93), mean (SD)^b^	46.9 (12.9)	56.3 (12.4)	64.3 (11.2)
White, N (%)^a^	5,710 (90.3)	3,673 (93.5)	2,678 (94.0)
Black, N (%)^a^	344 (5.4)	149 (3.8)	96 (3.4)
Other races, N (%)^a^	271 (4.3)	107 (2.7)	75 (2.6)
SES (0–1), mean (SD)^a,c^	0.5 (0.3)	0.5 (0.3)	0.6 (0.3)
Never smoked, N (%)^d^	3,080 (48.7)	1918 (48.8)	1,448 (50.9)
Former smoker, N (%)^d^	1867 (29.5)	1,443 (36.7)	1,144 (40.2)
Current smoker, N (%)^d^	1,377 (21.8)	568(14.5)	256 (9.0)
Any alcohol abuse, N (%)^a^	432 (6.8)	246 (6.3)	174 (6.1)
Any drug abuse, N (%)^a^	432 (6.8)	231 (5.9)	173 (6.1)
Obesity index (−3.8–5.8), mean (SD)^a,e^	0.0 (1.0)	0.0 (1.0)	0.0 (1.0)
Physical limitations index (−1.3–1.6), mean (SD)^e^	−0.2 (1.0)	0.1 (1.0)	0.3 (1.0)
Number of respondents	6,325	3,929	2,849

^a^Measured at baseline (Wave 1). ^b^As noted earlier, the sampling frame at Wave 1 targeted adults aged 25–74, but the age range of the final sample was 20–75 at Wave 1, 30–84 at Wave 2, and 39–93 at Wave 3. ^c^The SES index was converted to a percentile rank and rescaled to range from 0 (bottom percentile) to 1 (top percentile). ^d^Smoking status was updated at Waves 2 and 3, although in the main analysis, we treated smoking status as fixed at baseline. ^e^Standardized to have a mean of 0 and SD of 1 among the pooled sample across all three waves.

### 2.3. Analytic strategy

We used standard practices of multiple imputation to handle missing data (14, 15); see section 1.3 of Supplementary material for details. We fit a linear mixed model with an individual-level random intercept to account for intra-individual correlation and a robust variance estimator to correct for family-level clustering. In Model 1, we controlled only for demographic characteristics: sex, age (using a quadratic specification), race, smoking history, and an interaction between smoking history and age (linear and quadratic terms) to test whether the age pattern of physical limitations differs by smoking history.

Socioeconomic status is a potential confounder that affects both the propensity to adopt smoking and physical function. Thus, part of the association between smoking and physical limitations may be a result of smokers being more likely to have low socioeconomic status (rather than representing a causal effect of smoking). Model 2 adjusted for socioeconomic status, which was interacted with age (linear and quadratic terms).

Smoking also tends to be correlated with alcohol and drug abuse, which may be inversely associated with physical function. Confounding with substance abuse could inflate the apparent effect of smoking. In Model 3, we further adjusted for alcohol and drug abuse.

In contrast, an inverse correlation between smoking and obesity could offset some of the negative effect of smoking on physical function. Obesity is not really a confounder because it does not cause smoking, but rather is likely to be affected by smoking. The negative effect of smoking on obesity may benefit physical function. Therefore, we further adjusted for obesity in Model 4 because it could act as a suppressor. Controlling for obesity helps distinguish between any positive effect that smoking may have via reducing obesity from the adverse effect of smoking on physical function.

In auxiliary analyses, we tested additional measures of smoking duration, time since quitting smoking, smoking intensity, and pack-years. We compared models using a categorical specification, but found that the linear specification yielded the best model fit (i.e., lower Bayesian Information Criterion) for smoking duration, intensity, and pack-years. In the case of time since quitting smoking, a dichotomous variable indicating whether the respondent had quit smoking fewer than 5 years prior to baseline yielded the best fit. Net of smoking status, we found no evidence that the effect of these other smoking measures varied by age; consequently, we included only the main effect for those variables. Finally, we evaluated the sensitivity of the results to specifying smoking status as a time-varying covariate that was updated at Waves 2 and 3.

## 3. Results

The mean score on the physical limitations index was 0.3 SD higher for respondents who smoked at baseline (0.2) than for those who never smoked (−0.1) (Table 2). Supplementary Figure S1 shows a smoothed plot of physical limitations across age by baseline smoking status. Even at very young ages there were notable disparities in physical function by smoking status. For example, at age 30, physical limitations were nearly one-quarter of a SD higher for current smokers than for those who never smoked.

**Table 2:** Descriptive statistics for physical limitations and potential confounders by smoking status at baseline.

		Smoking status at baseline		
	Total	Never Smoked	Former smoker	Current smoker
Physical limitations index, mean (SD)^b^	0.0 (1.0)	−0.1 (1.0)	0.1 (1.0)	0.2 (1.00)
Female, %^a^	54.0	58.5	46.4	54.2
Age, mean (SD)	53.5 (14.2)	52.5 (14.6)	57.3 (13.7)	50.4 (12.9)
White, %^a^	92.0	91.0	93.9	92.0
Black, %^a^	4.5	5.5	3.2	4.0
Other races, %^a^	3.5	3.6	2.8	4.0
SES, mean (SD)^a^	0.5 (0.3)	0.6 (0.3)	0.5 (0.3)	0.4 (0.3)
Any alcohol abuse, %^a^	6.5	4.4	6.1	12.4
Any drug abuse, %^a^	6.4	4.4	5.0	13.5
Obesity index, mean (SD)^a,b^	0.0 (1.00)	0.0 (1.0)	0.2 (1.0)	−0.2 (1.0)
Number of observations^c^	13,103	6,575	3,919	2,609

^a^Measured at baseline (Wave 1). ^b^Standardized to have a mean of 0 and SD of 1 among the pooled sample. ^c^The analysis sample includes 6,325 respondents observed at Wave 1, 3,929 at Wave 2, and 2,849 at Wave 3 for a total of 13,103 observations.

Several potential confounders also differed substantially by smoking status. Compared with those who never smoked, current smokers had lower SES, were much more likely to report alcohol or drug abuse, and exhibited lower levels of obesity (Table 2). In contrast, former smokers had the highest average levels of obesity, perhaps because they gained weight when they quit smoking. Former smokers were also more likely to be male and tended to be older, on average, than never or current smokers.

Results from the demographic-adjusted regression model indicated that the age-related increase in physical limitations was steeper for respondents who smoked at baseline than for never smokers (Table 3, Model 1). That is, the effect of current smoking increased with age. Table 4 shows the estimated disparity in physical limitations at selected ages by baseline smoking status. When adjusted only for demographic characteristics (Model 1), the effect of smoking was apparent even at younger ages: at age 30, physical limitations were 0.24 SD higher for respondents who smoked at baseline relative to those who never smoked. That difference would be similar in magnitude to the difference between a respondent who scored 0.64 vs. 0.37 on the standardized physical limitation index. For example, among respondents observed at age 30, there was a current smoker who scored 0.64 on the physical limitations index and a never smoker who scored 0.37 on the index. Both of those respondents reported a little limitation on 4 physical tasks, but the current smoker also reported some limitation on a 5th task (which made his/her score about 0.25 SD higher than the never smoker).

**Table 3:** Coefficients (95% confidence intervals) from linear random intercept models predicting the physical limitations index.

Variable	Model 1	Model 2	Model 3	Model 4	Model 5
Female (vs. male)	0.265*** (0.223, 0.306)	0.226*** (0.185, 0.266)	0.240*** (0.200, 0.280)	0.270*** (0.232, 0.308)	0.286*** (0.248, 0.325)
Age^a^	0.271*** (0.255, 0.287)	0.297*** (0.266, 0.327)	0.308*** (0.277, 0.338)	0.286*** (0.256, 0.315)	0.285*** (0.256, 0.315)
Age squared^a^	0.031*** (0.024, 0.038)	0.001 (−0.013, 0.014)	−0.002 (−0.015, 0.012)	0.005 (−0.008, 0.018)	0.005 (−0.008, 0.018)
Black (vs. white)	0.171** (0.062, 0.279)	0.069 (−0.035, 0.172)	0.074 (−0.028, 0.177)	0.020 (−0.075, 0.114)	0.033 (−0.062, 0.127)
Other races (vs. white)	0.131* (0.028, 0.234)	0.106* (0.004, 0.209)	0.088 (−0.015, 0.190)	0.096 (−0.005, 0.197)	0.104* (0.003, 0.205)
SES^b,c^		−0.699*** (−0.785, −0.613)	−0.700*** (−0.786, −0.614)	−0.582*** (−0.664, −0.500)	−0.560*** (−0.642, −0.477)
Age^a^ × SES^b^		−0.049* (−0.094, −0.004)	−0.059* (−0.104, −0.014)	−0.055* (−0.099, −0.012)	−0.055* (−0.098, −0.012)
Age squared^a^ × SES^b^		0.050*** (0.030, 0.071)	0.053*** (0.032, 0.074)	0.050*** (0.030, 0.070)	0.050*** (0.030, 0.070)
Any alcohol abuse^b^			0.135*** (0.057, 0.212)	0.175*** (0.099, 0.250)	0.166*** (0.091, 0.242)
Any drug abuse^b^			0.333*** (0.249, 0.417)	0.356*** (0.276, 0.437)	0.353*** (0.273, 0.432)
Obesity index^b,d^				0.267*** (0.248, 0.286)	0.263*** (0.244, 0.282)
Former (vs. never smoked)^b,c^	0.126*** (0.069, 0.183)	0.085** (0.029, 0.141)	0.079** (0.024, 0.135)	0.054* (0.002, 0.107)	−0.087* (−0.155, −0.020)
Age^a^ × Former smoker^b^	0.023 (−0.008, 0.055)	0.025 (−0.006, 0.057)	0.027 (−0.005, 0.058)	0.022 (−0.009, 0.052)	0.023 (−0.008, 0.054)
Age squared^a^ × Former smoker^b^	−0.002 (−0.015, 0.011)	0.000 (−0.013, 0.014)	0.001 (−0.013, 0.014)	0.001 (−0.012, 0.014)	0.002 (−0.011, 0.015)
Current (vs. never smoked)^b,c^	0.393*** (0.326, 0.459)	0.264*** (0.197, 0.331)	0.226*** (0.159, 0.293)	0.300*** (0.237, 0.364)	0.177*** (0.098, 0.256)
Age^a^ × Current smoker^b^	0.052** (0.020, 0.084)	0.049** (0.017, 0.082)	0.057*** (0.024, 0.090)	0.059*** (0.027, 0.090)	0.056*** (0.024, 0.087)
Age squared^a^ × Current smoker^b^	−0.013 (−0.031, 0.005)	−0.004 (−0.022, 0.014)	−0.005 (−0.023, 0.013)	−0.006 (−0.023, 0.012)	−0.004 (−0.022, 0.013)
Quit smoking <5 years ago^b^					0.164*** (0.072, 0.257)
Packs of cigarettes per day^b^					0.093*** (0.059, 0.127)
Constant	−0.417*** (−0.458, −0.376)	0.011 (−0.056, 0.079)	−0.016 (−0.083, 0.051)	−0.107** (−0.171, −0.044)	−0.130*** (−0.194, −0.066)
SD of random intercept	0.659*** (0.643, 0.676)	0.637*** (0.620, 0.654)	0.631*** (0.614, 0.648)	0.572*** (0.554, 0.590)	0.569*** (0.551, 0.587)
SD of the residual	0.607*** (0.594, 0.619)	0.606*** (0.594, 0.619)	0.606*** (0.594, 0.618)	0.607*** (0.594, 0.619)	0.607*** (0.551, 0.587)

The analysis sample includes 13,103 observations for 6,325 respondents observed at Wave 1, of whom 3,929 were also observed at Wave 2 and 2,849 at Wave 3. ^a^Age is centered at 50 and divided by 10 (i.e., the age coefficient represents the effect of 10 age years). ^b^Measured at baseline (Wave 1). ^c^This coefficient represents the effect at age 50. The corresponding effect at any other age X can be obtained as follows: $\beta^{Z}+\frac{\left(X-50\right)}{10}\times \beta^{Age\times Z}+{\left(\frac{\left(X-50\right)}{10}\right)}^{2}\times \beta^{Age^{2}\times Z}$, where $\beta^{Z}$ is the main effect for variable Z, $\beta^{Age\times Z}$ is the interaction between linear age (i.e., $\frac{\left(X-50\right)}{10}$) and Z, and $\beta^{Age^{2}\times Z}$ is the interaction between age-squared (i.e.,$\;{\left(\frac{\left(X-50\right)}{10}\right)}^{2})\;$ and Z. For example, the difference between current and never smokers at age 30 (i.e., $\frac{\left(30-50\right)}{10}=2$) based on Model 1 would be: 0.393−2×0.052+4×(−0.013)=0.24. ^d^Standardized to have a mean of 0 and SD of 1 among the pooled sample. ***p < 0.001, **p < 0.01, *p < 0.05.

**Table 4:** Estimated disparity (95% confidence interval) in physical limitations (standardized) at selected ages by smoking subgroups.

	At age					
	30	40	50	60	70	80
Model 1: demographic-adjusted						
Former vs. never smoker	0.07 (−0.03, 0.17)	0.10** (0.04, 0.16)	0.13*** (0.07, 0.18)	0.15*** (0.09, 0.21)	0.16*** (0.10, 0.23)	0.18*** (0.09, 0.27)
Current vs. never smoker	0.24*** (0.14, 0.33)	0.33*** (0.27, 0.39)	0.39*** (0.33, 0.47)	0.43*** (0.36, 0.50)	0.44*** (037, 0.53)	0.43*** (0.30, 0.57)
Current vs. former smoker	0.16* (0.04, 0.29)	0.23*** (0.15, 0.30)	0.27*** (0.19, 0.34)	0.28*** (0.21, 0.36)	0.28**** (0.20, 0.36)	0.25*** (0.11, 0.39)
Model 4: fully-adjusted						
Former vs. never smoker	0.02 (−0.08, 0.11)	0.03 (−0.03, 0.09)	0.05* (0.00, 0.11)	0.08** (0.02, 0.13)	0.10*** (0.05, 0.16)	0.13*** (0.05, 0.21)
Current vs. never smoker	0.16** (0.06, 0.26)	0.24*** (0.17, 0.30)	0.30*** (0.24, 0.36)	0.35*** (0.29, 0.42)	0.40*** (0.32, 0.47)	0.43*** (0.30, 0.56)
Current vs. former smoker	0.15* (0.02, 0.27)	0.20*** (0.13, 0.27)	0.25*** (0.18, 0.31)	0.28*** (0.21, 0.35)	0.29*** (0.21, 0.37)	0.30** (0.16, 0.43)
Model 5: fully-adjusted						
Former (5+ years) light smoker vs. never smoker	−0.08 (−0.18, 0.02)	−0.06 (−0.13, 0.00)	−0.04 (−0.10, 0.02)	−0.02 (−0.08, 0.04)	0.01 (−0.05, 0.07)	0.04 (−0.05, 0.13)
Former (5+ years) heavy smoker vs. never smoker	0.06 (−0.05, 0.17)	0.08* (0.01, 0.15)	0.10** (0.04, 0.16)	0.12*** (0.06, 0.18)	0.15*** (0.09, 0.21)	0.18*** (0.09, 0.27)
Former (<5 years) light smoker vs. never smoker	0.08 (−0.03, 0.20)	0.10* (0.01, 0.20)	0.12* (0.03, 0.22)	0.15** (0.05, 0.25)	0.18** (0.07, 0.28)	0.21** (0.09, 0.33)
Former (<5 years) heavy smoker vs. never smoker	0.22*** (0.10, 0.34)	0.24*** (0.15, 0.34)	0.26*** (0.17, 0.36)	0.29*** (0.19, 0.39)	0.32*** (0.21, 0.42)	0.35*** (0.23, 0.46)
Current light smoker vs. never smoker	0.10 (0.00, 0.19)	0.16*** (0.10, 0.23)	0.22*** (0.15, 0.29)	0.27*** (0.20, 0.35)	0.32*** (0.24, 0.40)	0.35*** (0.22, 0.49)
Current heavy smoker vs. never smoker	0.24*** (0.13, 0.34)	0.30*** (0.24, 0.37)	0.36*** (0.30, 0.43)	0.41*** (0.35, 0.48)	0.46*** (0.38, 0.54)	0.49*** (0.36, 0.63)

Estimates are based on the regression models shown in Table 3. A light smoker is defined as someone who smokes 10 cigarettes (i.e., half a pack) per day and a heavy smoker denotes 40 cigarettes (i.e., 2 packs) per day. *p < 0.05, **p < 0.01, ***p < 0.001.

By age 70, the differential between current and never smokers increased to 0.44 SD (Table 4, Model 1). Even former smokers exhibited a non-negligible effect relative to never smokers, at least above age 40: the differential between those who had quit smoking by baseline and never smokers was 0.10 SD at age 40 but grew to 0.18 SD at age 80.

Further adjustment for SES attenuated the smoking differentials. At age 50, the difference between respondents who smoked at baseline and never smokers was reduced from 0.39 SD in Model 1 to 0.26 SD after adjustment for SES in Model 2 (Table 3). The differential between former and never smokers at age 50 also diminished somewhat (from 0.13 SD in Model 1 to 0.09 SD in Model 2).

Adjusting for alcohol and drug abuse in Model 3 further reduced the smoking differentials (i.e., confounding between smoking and other substance abuse was inflating the apparent effect of smoking). At age 50, the differential between current and never smokers was reduced to 0.23 SD.

In Model 4, we added obesity, which increased the differential between current and never smokers (from 0.23 SD at age 50 in Model 3 to 0.30 SD in Model 4), suggesting that lower levels of obesity among smokers were offsetting some of the adverse effects of smoking. In contrast, controlling for obesity further attenuated the difference between former and never smokers (from 0.08 SD at age 50 in Model 3 to 0.05 SD in Model 4), which implies that higher levels of obesity among former smokers were inflating the apparent effect of former smoking.

**Figure 1:**
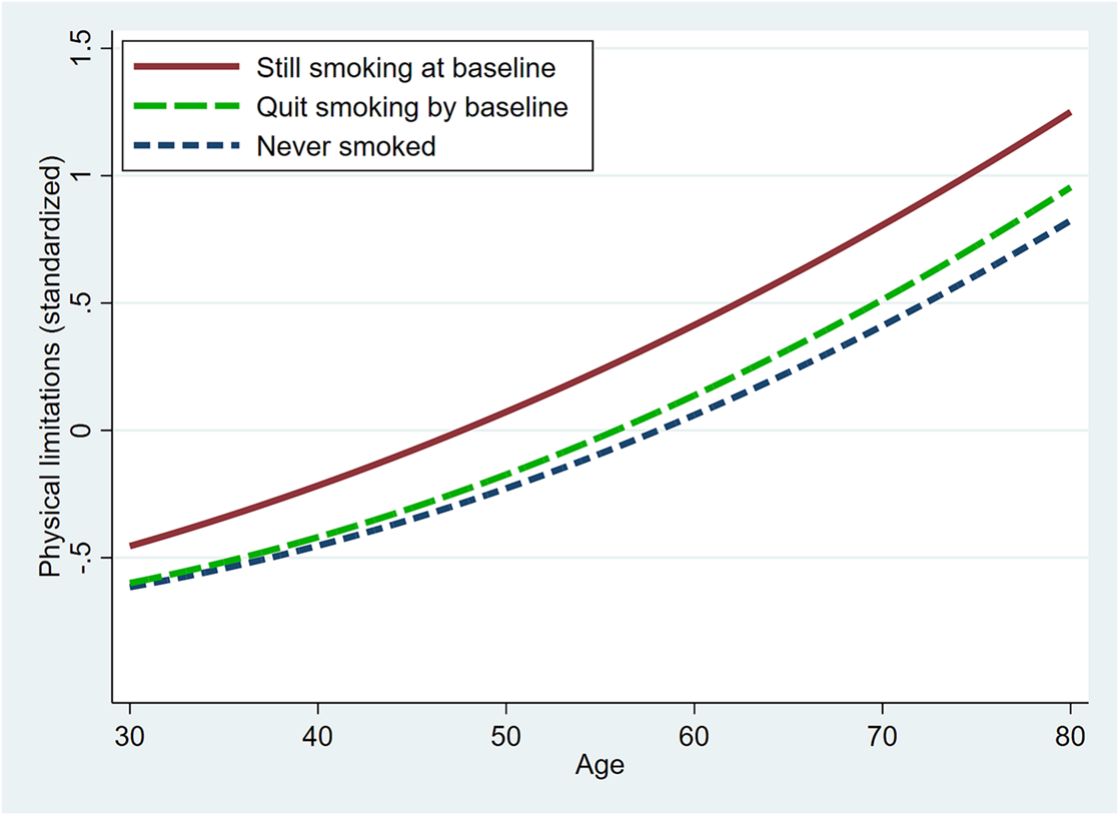
Model-based predicted physical limitations across age by smoking status at baseline. Predicted values are based on Model 4 (Table 3) with covariates set to the sample means.

In the fully-adjusted model (Model 4), current smokers had significantly more physical limitations than never smokers even at ages as young as 30. The differential between respondents who smoked at baseline and never smokers rose from 0.16 SD at age 30 to 0.43 SD at age 80 (Table 4). However, there was a much smaller differential between former and never smokers (e.g., 0.13 SD at age 80). Figure 1 shows the predicted values across age by smoking status at baseline based on the fully-adjusted model. Smokers exhibited a steeper age-related increase in physical limitations than never smokers. Thus, the disparities in physical limitations by smoking status widened with age but were evident even at relatively young ages.

### 3.1. Sensitivity analyses

Auxiliary models revealed that, net of smoking status, smoking duration was not significantly associated with physical limitations (Supplementary Table S1, Model 1). However, former smokers who quit fewer than 5 years prior to baseline exhibited a level of physical limitations that was 0.18 SD higher than long-term former smokers (Model 2). Smoking intensity (Model 3) and pack-years (Model 4) were both associated with more physical limitations, but the association was stronger for intensity than pack-years.

**Figure 2:**
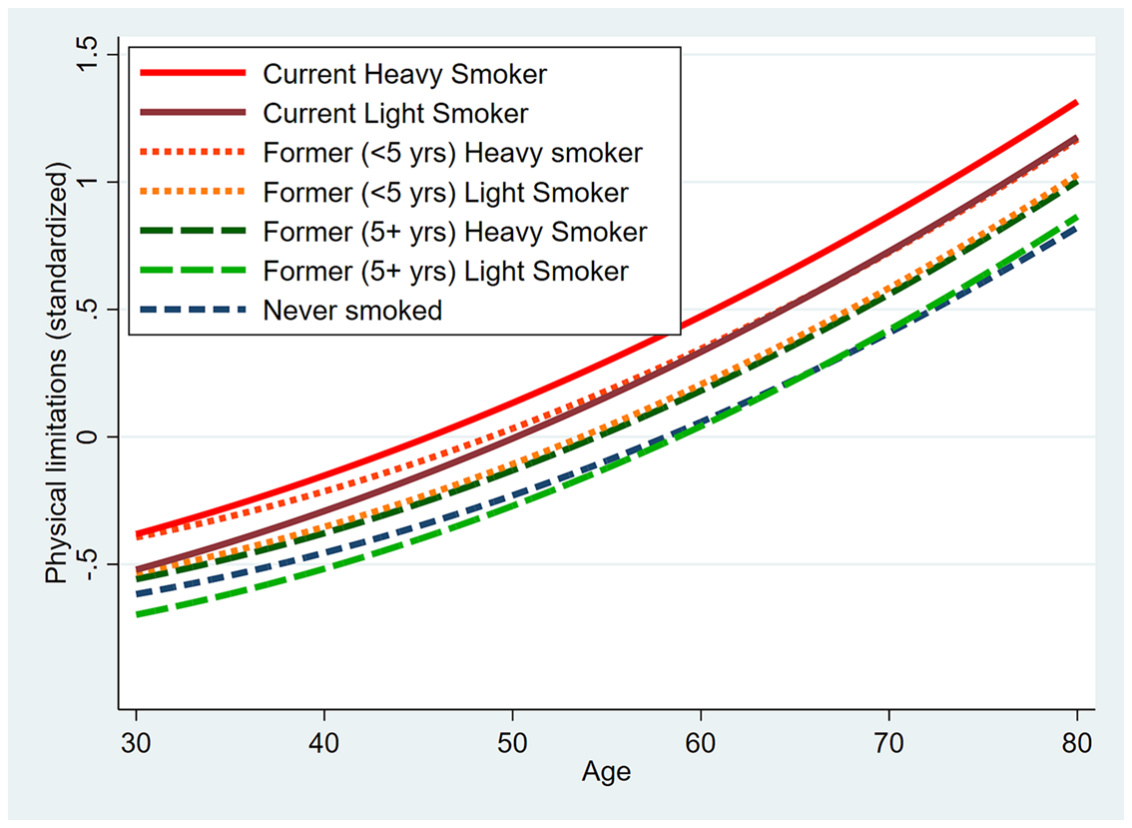
Model-based predicted physical limitations across age by smoking history and selected levels of intensity. Predicted values are based on Model 5 (Table 3) with covariates set to the sample means.

Therefore, we estimated an additional model that included the dichotomous indicator for former smokers who quit less than 5 years prior to baseline as well as smoking intensity (Tables 3, 4, Model 5). Figure 2 shows the predicted values across age for selected levels of baseline smoking status and intensity. Current heavy smokers exhibited the highest levels of physical limitations (e.g., 0.24 SD higher than never smokers at age 30 and 0.49 SD higher at age 80; Table 4, Model 5). A difference of 0.49 SD would be similar in magnitude to the difference between a respondent who scored 1.61 vs. 1.14. For example, among respondents observed at age 80, there was a current heavy smoker (i.e., 2 packs/day) who scored 1.61 on the physical limitations index and a never smoker who scored 1.14 on the index. The current heavy smoker reported some limitation on one task and a lot of limitation on seven tasks, whereas the never smoker reported a little limitation on one task, some limitation on four tasks and a lot of limitation on only one task. Consequently, the current heavy smoker scored about 0.5 SD higher than the never smoker on the physical limitations index.

At younger ages, heavy smokers who quit recently appeared to fare worse than current light smokers and not much better than current heavy smokers (Table 4, Model 5). In contrast, former light smokers who quit at least 5 years prior to baseline did not differ significantly from never smokers.

When smoking status was treated as a time-varying covariate (Supplementary Table S2), the differentials between current and never smokers were smaller that when smoking was measured only at baseline (Table 4, Model 4), whereas the differentials between former and never smokers were generally larger, particularly at older ages. Consequently, the differences between a current and former smoker were smaller. When smoking status was treated as a time-varying covariate, we suspect the effect of smoking may have been under-estimated (because some smokers who experienced health problems quit smoking as a result), while the differential between former and never smokers was over-estimated (because the “former smoker” group is contaminated with some respondents who quit smoking because they experienced health problems). Thus, we believe it is better to measure smoking status only at baseline, which helps avoid the problem that ill health may affect the decision to quit smoking.

## 4. Discussion

We know smoking is bad for our health, but these results reveal that differences in physical limitations by smoking history are evident even at ages as young as 30. Physical function has important implications for quality of life, productivity, health care costs, disability, and the need for long-term care. Physical limitations that emerge early in life are likely to have an especially large impact because they can jeopardize health for decades of remaining life.

Our results suggest that smoking differentials in physical limitations widen across the age range from 25 to 80. In contrast, Peeters et al. (9) seemed to find that smoking differentials were largest around age 65 among Australian women, but they did not formally test an interaction between age and smoking across the full age range of the sample (18–91).

We find smoking intensity is more strongly associated with physical function than duration or pack-years. That result is consistent with Dai et al. (16) argument that intensity is more important than smoking duration for most health outcomes other than cancer.

Our effect sizes of one-quarter SD (at younger ages) to half a SD (at older ages) would be considered small to medium (17, 18). Many of the other outcomes for which the effects of smoking have been evaluated are dichotomous (e.g., mortality, various chronic conditions, physical impairment); thus, effect size is generally evaluated in terms of relative ratios rather than in SD (i.e., Z-score) units (1, 3, 16).

Matthay et al. (17) provided a correspondence for the magnitude of effect size across different measures (see Table 2). For example, a small standardized mean difference (0.2–0.4) corresponds with a relative risk (when the risk of the outcome is 1% among unexposed) of 1.4–2.0, whereas a medium standardized mean difference (0.5–0.7) corresponds with a relative risk of 2.4–3.5, and a large standardized mean difference (0.8–1.1) corresponds with a relative risk greater than 4.1. Thus, we consider an increased risk ((RR−1)×100) of 40–100% (i.e., *RR* 1.4–2.0) to be a small effect, 101–250% to be a medium effect, and greater than 250% to be a large effect.

By that standard, the effect of cigarette smoking on all-cause mortality among men—70% increased risk according to the Surgeon General’s report (19)—would be a small effect. A meta-analysis (16) of the effects of smoking across 36 health outcomes reported that the average increased risk associated with smoking was less than 40% for 27 outcomes (including ischemic heart disease, stroke, and type 2 diabetes), 40–100% for five outcomes (e.g., COPD), 101–250% for three outcomes (e.g., lung cancer), and above 250% for only one outcome (laryngeal cancer). In comparison, our effect sizes would seem to be as large or larger than the effects for many health outcomes.

We are aware of a few other studies that used a continuous measure of physical function, but none of them quantified their effect size in terms of SD units. Based on the information provided regarding the SD of the outcome, we are able to infer that one of those studies (2) reported a much larger effect of smoking than ours (more than 2 SD), while another (8) reported a much smaller effect (0.12 SD for men, 0.03 SD for women). We do not understand how the effect size was so large in the first study (2), but their outcome was a count of the number of physical limitations, which gives more weight to high levels of limitations than our log-transformed index. Absolute scoring gives equal weight to each increment, whereas log-transformation assumes that each additional increment has less impact (i.e., effect is relative). Thus, log-transformation tends to reduce the differentials at high levels of limitations (i.e., which is more common at older ages). As Long and Pavalko (11) point out, it is probably unrealistic to assume that each additional limitation has the same impact. Thus, they find that log-transformed versions of the physical function index tend to perform better than untransformed versions. We suspect that the effect size in the second study (8) is smaller than ours because they included additional covariates (e.g., chronic diseases, pain) that may mediate the effect of smoking on physical function.

One study limitation is that although we controlled for key potential confounders (i.e., SES, substance abuse, obesity), other omitted variables that differ by smoking status may also affect physical function. Thus, unobserved heterogeneity could bias our estimates of the smoking effect. Second, some respondents may have quit smoking prior to baseline because of health problems, which could inflate physical limitations among former smokers relative to current smokers. Third, mortality selection could reduce the smoking differentials at the oldest ages because smokers were likely to die earlier in life than non-smokers. Indeed, 23% of those who reported smoking at baseline died before Wave 3 (median age at death = 68.5) versus 21% of former smokers (median age at death = 77.0) and only 11% of those who never smoked (median age at death = 78.8). Many smokers with serious health problems may have already died by age 70; survivors may represent a select group of the healthiest smokers. Finally, observational studies such as this one may underestimate the effect of smoking because of the long lag between smoking initiation and the health consequences and imperfect retrospective recall by the respondent.

Smoking probably will not kill you at young age, but it may compromise your physical function long before it kills you. Just do not do it.

## Data availability statement

The original contributions presented in the study are included in the article/Supplementary material, further inquiries can be directed to the corresponding author.

## Ethics statement

The studies involving humans were approved by the Educational and Social/Behavioral Science Institutional Review Board at University of Wisconsin, Madison. The studies were conducted in accordance with the local legislation and institutional requirements. The participants provided their written informed consent to participate in this study.

## Author contributions

DG: Conceptualization, Data curation, Formal analysis, Investigation, Methodology, Software, Visualization, Writing – original draft. MW: Conceptualization, Funding acquisition, Investigation, Project administration, Writing – review & editing.

## Footnotes

^1^Although the sampling frame targeted adults aged 25–74, the final sample included a few respondents aged 20–24 (*N* = 15) or aged 75 (*N* = 4) at the baseline phone interview.
