# Supplementary material for: Smoking may compromise physical function long before it kills you
Source: Front Public Health. 2023 Nov 8;11:1261102. doi: 10.3389/fpubh.2023.1261102 (PMC10666746; doi:10.3389/fpubh.2023.1261102)
Supplement: Supplementary file 1 [file Table_1.docx]

Supplementary Material

# Supplemental Methods

## Data

National random digit dialing with oversampling of older people and men was used to select the main sample (*N*=3,487) and a sample of twin pairs (*N*=1,914) at Wave 1. The study also included a random sample of siblings of individuals in the main sample (*N*=950) and oversamples from five metropolitan areas in the U.S. (*N*=757). The response rate for the phone interview ranged from 60% for the twin subsample to 70% for the main sample. Among those who completed the phone interview (*N*=7,108), 6,325 (89%) also completed mail-in self-administered questionnaires. Brim et al. (1) compared the sociodemographic characteristics of the Wave 1 self-administered questionnaire sample with the corresponding distributions from the October 1995 Current Population Survey (CPS). The sample had a lower percentage of women (51.5% vs. 52.5% in the CPS), a higher percentage of Whites (87.7% vs. 84.1%, respectively), fewer participants at younger (e.g., 20.5% aged 25-34 vs. 25.4% in the CPS) and older (e.g., 11.2% aged 65-74 vs. 12.4% in the CPS) ages, a lower percentage of individuals with 12 or fewer years of education (39.2% vs. 49.5%, respectively), and a lower percentage of married persons (64.0% vs. 67.4%, respectively).

## Measures

### Race

Respondents were asked, “*What race do you consider yourself to be?*” We retained the first two response categories (i.e., Black and/or African American; White), but combined the remaining categories (i.e., Asian or Pacific Islander; multiracial; Native American or Aleutian Islander/Eskimo; other) into a group labeled “other races.” We did not include ethnicity because the 1995-96 wave of MIDUS did not ask respondents to report their ethnicity.

### Socioeconomic status

Education (of the respondent and of the spouse/partner, if applicable) was based on degree completion with 12 response categories ranging from less than 8th grade to completion of a professional degree (e.g., PhD, MD, JD, etc.). The occupational socioeconomic index (SEI) was created by Hauser and Warren (2) based on the three-digit 1980 census occupational codes; scores range from 7.1 (shoe machine operator) to 80.5 (physician). Annual household income included all sources of income for the respondent, spouse/partner, and all other family members living in the household. Total net assets were reported for the respondent and spouse/partner combined. (See next section for more details regarding income and assets.)

### Household Income and Assets

Income from each source (i.e., wages/salary, social security, government assistance, and all other sources such as pensions, investments, child support, or alimony) was reported in categories, which we recoded to the mid-point of the range within each category. Income from each source was top-coded at $200,000 (except government assistance, which was top-coded at $50,000). We recoded top-coded values to the harmonic mean of a Pareto distribution. As suggested by von Hippel (3), we compute the harmonic mean of a Pareto distribution with $\alpha$ equal to the maximum of one or $\frac{\ln\left( n_{B-1}+n_{B} \right)-ln(n_{B})}{\ln\left( l_{B} \right)-ln(l_{B-1})}$, where $n_{B}$ is the number of cases in the top category; $n_{B-1}$ is the number of cases in the penultimate category; $l_{B}$ is the lower bound of the top category; and $l_{B-1}$ is the lower bound of the penultimate category. Restricting $\alpha$ to a minimum of one ensures that the value of the top category is no greater than twice the lower bound of that category. We then summed across all sources to compute total income. We were unable to make an equivalence adjustment based on household size and composition because MIDUS did not collect that information at Wave 1.

Assets were also reported in categories and recoded to the mid-point of each range, with those who reported themselves to be in debt coded as a negative number. The top category ($1,000,000+) was recoded to the harmonic mean of a Pareto distribution as described above for income.

For interviews conducted in 1996, we converted income and assets to 1995 dollars using the Consumer Price Index (CPI) provided by the Bureau of Labor Statistics (<https://data.bls.gov/cgi-bin/cpicalc.pl>). The median month among interviews completed in 1995 was April (which is treated as the reference), and the median month for those conducted in 1996 was July. Thus, the multiplier was 0.97 for data collected in 1996.

### Alcohol Abuse

Our measure of alcohol abuse, which was based on four items from the Michigan Alcohol Screening Test (4), has been used in previous studies (5,6).

### Drug Abuse

Our measure of drug abuse, which was based on the CIDI-SF Drug Dependence Scale (7), has also been used in prior studies (5,8,9). Unlike the CIDI-SF, which asked about the specified drug-related problems if the respondent used any drug, MIDUS asked these questions only if the respondent reported misuse of a drug (i.e., use of sedatives, tranquilizers, amphetamines, prescription painkillers, inhalants, marijuana/hashish, cocaine/crack/free base, hallucinogens, heroin, or prescription anti-depressants) “on your own”—that is, “without a doctor’s prescription, in larger amounts that prescribed, or for a long period than prescribed.”

### Obesity

Body mass index (BMI) was calculated based on self-reported height and weight. BMI one year prior to the baseline was based on a retrospective question about the respondent’s weight one year ago. The subjective question about the respondent weight included five response categories, ranging from “very underweight” to “very overweight.” In addition to well-known sex differences in waist and hip circumference, which are independent of obesity, the subjective evaluation of being overweight also differed by sex. Auxiliary analyses showed that men were far less likely to report themselves as being “overweight” than women even after controlling for BMI.

## Multiple Imputation

Among the 6,325 respondents included in the analysis, the predictors with the highest percentage of missing data were household income (10%), wealth (10%), hip circumference (8%), and waist circumference (7%). For the outcome variable (index of physical limitations), the ability to walk one block has the highest level of missing data (0.6% at Wave 1, 1.6% at Wave 2, 2.2% at Wave 3). All other analysis variables were missing for less than 5% of respondents. We used the “ice” command in Stata 16.1 (10) to perform multiple imputation. For the multiple imputation process, we used information for all the analysis variables as well vital status on December 31, 2019, and baseline measures of marital status, employment status, wages/salary income of the respondent and of the spouse/partner, perceived economic distress, and reported difficulty bathing or dressing.

For continuous variables, departures from normality may result in implausible imputations when using the default draw method. Income and assets had a skewed distribution. Prior to imputation, we applied an inverse hyperbolic sine transformation (11) to the income and asset variables. To ensure that imputed values were within the range of observed values, we used prediction matching for those variables and several others for which imputation generated out of range values (i.e., age, education, occupational SEI score, wages/salary income, and rating of current financial situation).

We used an ordered logit model for imputation of ordinal variables (e.g., physical limitations). We performed five imputations and then used the “mim” prefix command to re-estimate the model for each imputation and combine the five sets of estimates using Rubin’s rules (12).

# Supplementary Figure

Figure S1. Smoothed plot of physical limitations across age by smoking status at baseline.

Note: Values are plotted with local mean smoothing—also known as the Nadaraya-Watson estimator (Nadaraya, 1964; Watson, 1964)—using the lpolyci command in Stata 16.1. (10) Smoking status is measured only at Wave 1. Physical limitations are measured at each wave (1, 2, and 3) for which the respondent completed the self-administered questionnaires. Thus, there are up to three observations per respondent (e.g., someone may have been observed at age 25 at Wave 1, at age 34 at Wave 2, and at age 43 at Wave 3). We standardized physical limitations based on the pooled distribution across all three waves. We plot the values only for the age range 30-80 because the sample comprises few respondents younger than 30 (particularly for former smokers) or older than 80 (particularly for current smokers). These analyses do not adjust for sex, race, SES, alcohol/drug abuse, or obesity.[Note: In these smoothed plots (which do not take into account multiple observations per R), the age patterns look more linear than they do when we fit a random intercept model controlling for age (quadratic), smoking, and the interaction between age and smoking (see ~\2023-06-08-Age Function\ Age-function-physical limitations-by-smoking status.docx). Also, there appears to be more convergence between former and never smokers at the oldest ages in the random intercept model.]

# Supplementary Tables

**Table S1. Linear random intercept models predicting the physical limitations index using various smoking-related measures**

| Variable | Model 1 | Model 2 | Model 3 | Model 4 |
| --- | --- | --- | --- | --- |
| Female (vs. Male) | 0.270*** | 0.269*** | 0.288*** | 0.283*** |
| Age^a^ | 0.286*** | 0.285*** | 0.286*** | 0.284*** |
| Age squared^a^ | 0.005 | 0.005 | 0.005 | 0.005 |
| Black (vs. White) | 0.019 | 0.017 | 0.036 | 0.028 |
| Other races (vs. White) | 0.096 | 0.091 | 0.109* | 0.104* |
| SES^b,c^ | -0.583*** | -0.571*** | -0.569*** | -0.572*** |
| Age^a^ x SES^b^ | -0.056* | -0.055* | -0.056* | -0.053* |
| Age squared^a^ x SES^b^ | 0.050*** | 0.050*** | 0.050*** | 0.050*** |
| Any alcohol abuse^b^ | 0.175*** | 0.172*** | 0.168*** | 0.170*** |
| Any drug abuse^b^ | 0.356*** | 0.355*** | 0.354*** | 0.358*** |
| Obesity index^b,d^ | 0.267*** | 0.268*** | 0.262*** | 0.264*** |
| Former smoker (vs. Never smoked)^b,c^ | 0.061 | 0.015 | -0.057 | 0.006 |
| Age^a^ x Former smoker^b^ | 0.023 | 0.031* | 0.014 | 0.010 |
| Age squared^a^ x Former smoker^b^ | 0.001 | -0.000 | 0.003 | 0.002 |
| Current smoker (vs. Never smoked)^b,c^ | 0.311*** | 0.303*** | 0.169*** | 0.216*** |
| Age^a^ x Current smoker^b^ | 0.061*** | 0.059*** | 0.056*** | 0.043* |
| Age squared^a^ x Current smoker^b^ | -0.006 | -0.006 | -0.004 | -0.004 |
| Smoking duration^b,d^ | -0.006 |  |  |  |
| Quit smoking < 5 years ago^b,e^ |  | 0.181*** |  |  |
| Cigarettes per day^b,d^ |  |  | 0.081*** |  |
| Pack-years^b,d^ |  |  |  | 0.055*** |
| Constant | -0.111** | -0.112*** | -0.066* | -0.089** |
| SD of random intercept | 0.572*** | 0.571*** | 0.569*** | 0.569*** |
| SD of the residual | 0.607*** | 0.606*** | 0.607*** | 0.607*** |

^a^ Age is centered at 50 and divided by 10 (i.e., the age coefficient represents the effect of 10 age years).

^b^ Measured at baseline (Wave 1).

^c^ This coefficient represents the effect at age 50. The corresponding effect at any other age $X$ can be obtained as follows: $\beta^{Z}+\frac{\left( X-50 \right)}{10}\times\beta^{Age \times Z}+\left( \frac{\left( X-50 \right)}{10} \right)^{2}\times\beta^{{Age}^{2} \times Z}$, where $\beta^{Z}$ is the main effect for variable $Z$, $\beta^{Age \times Z}$ is the interaction between linear age (i.e., $\frac{\left( X-50 \right)}{10}$) and $Z$, and $\beta^{{Age}^{2} \times Z}$ is the interaction between age-squared (i.e.,$\left( \frac{\left( X-50 \right)}{10} \right)^{2})$and $Z$. For example, the difference between current and never smokers at age 30 (i.e., $\frac{\left( 30-50 \right)}{10}=2$) based on Model 1 would be: $0.393-2\times0.052+4\times(-0.014)=0.23$.

^d^ Standardized to have a mean of 0 and SD of 1 among the pooled sample.

^e^ The reference group includes long-term former smokers (i.e., quit at least 5 years prior to baseline) as well as never and current smokers. The coefficient indicates the difference between someone who quit smoking fewer than 5 years before baseline and a long-term former smoker (represented by the main effect for former smokers).

*** *p*<0.001, ** *p*<0.01, * *p*<0.05

Table S2. Estimated disparity in physical limitations (standardized) at selected ages by smoking status as a time-varying covariate, fully-adjusted

|  | At Age: | | | | | |
| --- | --- | --- | --- | --- | --- | --- |
|  | 30 | 40 | 50 | 60 | 70 | 80 |
| Former vs. Never Smoker | 0.00 | 0.06* | 0.11*** | 0.15*** | 0.18*** | 0.21*** |
| Current vs. Never Smoker | 0.14** | 0.21*** | 0.26*** | 0.28*** | 0.28*** | 0.25** |
| Current vs. Former Smoker | 0.14* | 0.16*** | 0.16*** | 0.14*** | 0.10* | 0.05 |

* *p* < 0.05, ** *p* < 0.01, *** *p* < 0.001

Note: These estimates are based on regression model with the same specification as Model 4 in Table 3, except that smoking status is treated as a time-varying covariate that is updated at Waves 2 and 3.

# References

1. Brim OG, Baltes PB, Bumpass LL, Cleary PD, Featherman DL, Hazzard WR, et al. National Survey of Midlife Development in the United States (MIDUS 1), 1995-1996: Documentation of Post-Stratification Weights Created at MIDUS 1. Inter-university Consortium for Political and Social Research [distributor], Version 19 [Internet]. 2019. Available from: https://doi.org/10.3886/ICPSR02760.v19

2. Hauser RM, Warren JR. Socioeconomic Indexes for Occupations: A Review, Update, and Critique. Sociological Methodology. 1996;27(1):177–298.

3. von Hippel PT, Scarpino SV, Holas I. Robust estimation of inequality from binned incomes. Sociological Methodology. 2016;46(1):212–51.

4. Selzer ML. The Michigan alcoholism screening test: the quest for a new diagnostic instrument. AmJPsychiatry. 1971;127(12):1653–8.

5. Glei DA, Weinstein M. Drug and alcohol abuse: the role of economic insecurity. AmJHealth Behav. 2019;43(4):838–53.

6. Ransome Y, Slopen N, Karlsson O, Williams DR. The association between alcohol abuse and neuroendocrine system dysregulation: race differences in a national sample. Brain, Behavior, and Immunity. 2017 Nov;66:313–21.

7. Kessler RC, Andrews G, Mroczek D, Ustun TB, Wittchen HU. The World Health Organization Composite International Diagnostic Interview Short Form (CIDI-SF). Int J Methods Psychiatr Res. 1998;7(4):171–85.

8. Glei DA, Weinstein M. Mental health, pain, and risk of drug misuse: A nationwide cohort study. Addict Behav. 2020 Oct;109:106467.

9. Glei DA, Stokes A, Weinstein M. Changes in mental health, pain, and drug misuse since the mid-1990s: Is there a link? Soc Sci Med. 2020;246:112789.

10. StataCorp. Stata: Release 16. Statistical Software. College Station, TX: StataCorp LLC; 2019.

11. Killewald A, Pfeffer FT, Schachner JN. Wealth inequality and accumulation. Annu Rev Sociol. 2017 Jul;43:379–404.

12. Royston P, Carlin JB, White IR. Multiple imputation of missing values: new features for mim. Stata Journal. 2009;9(2):252–64.
